# Effects of Ramadan Fasting on Extracorporeal Photopheresis Outcomes

**DOI:** 10.7759/cureus.47612

**Published:** 2023-10-24

**Authors:** Yandy M Castillo-Aleman, May A Martinez, Yendry Ventura-Carmenate, Carlos A Villegas-Valverde, Antonio A Bencomo-Hernandez, Rene A Rivero Jimenez, Fatema M Al-Kaabi

**Affiliations:** 1 Immunology, Abu Dhabi Stem Cells Center, Abu Dhabi, ARE; 2 Nursing, Abu Dhabi Stem Cells Center, Abu Dhabi, ARE; 3 Immunology Laboratory, Abu Dhabi Stem Cells Center, Abu Dhabi, ARE; 4 Stem Cells Procesing Laboratory, Abu Dhabi Stem Cells Center, Abu Dhabi, ARE; 5 Hematology, Abu Dhabi Stem Cells Center, Abu Dhabi, ARE

**Keywords:** health education, treatment outcome, religious fasting, fasting, extracorporeal photopheresis

## Abstract

Although there are many studies on the impact of Ramadan fasting on health in the medical literature, the effects have not been explored in Muslim patients undergoing extracorporeal photopheresis (ECP). This report aimed to describe the potential effects of Ramadan fasting on ECP treatment outcomes. Patients undergoing ECP were prospectively evaluated before and during the month of Ramadan 1443 AH (2022 AD) at the Abu Dhabi Stem Cells Center (ADSCC), United Arab Emirates. The following ECP outcomes were assessed: treatment completion, adverse events reported, body mass index (BMI), and laboratory test results, including complete blood count (CBC), C-reactive protein (CRP), and other systemic immune-inflammatory biomarkers (SIIBs). No statistically significant differences were found in most of the variables analyzed in the three patients who underwent ECP before and during the holy month. Two non-fasting patients were not able to complete the Ramadan ECP schedule, and one fasting patient experienced a vascular access event during his first procedure in Ramadan. These findings suggest that fasting during Ramadan could add further risk factors and develop serious complications related to the ECP treatment. Therefore, we suggest that fasting should be avoided during photopheresis treatment, and we provided recommendations to achieve the best possible clinical outcomes.

## Introduction

Observing Ramadan fasting is one of the five pillars of Islam. All healthy Muslims who have reached puberty are obliged to fast during the Islamic holy month, although exceptions are made for pregnant or breastfeeding women, Muslims who are traveling or sick, and older people. While fasting, adult Muslims abstain from sexual activities, eating, drinking, and smoking during daylight hours.

For worshippers with specific diseases, some societies, such as the International Diabetes Federation (IDF) and the Diabetes and Ramadan (DaR) International Alliance, have recommended improving awareness and management of the relevant clinical conditions during Ramadan [1]. Other groups, such as the British Islamic Medical Association (BIMA) [2], recently provided recommendations for healthcare professionals who counsel fasting patients with miscellaneous conditions. Although there are many publications related to the clinical effects of Ramadan fasting, it has not been explored in Muslim patients undergoing extracorporeal photopheresis (ECP).

ECP was initially approved for the palliative treatment of advanced cutaneous T-cell lymphoma, and it has become a new therapeutic option for other conditions, including graft versus host disease (GvHD), solid organ transplant rejection, and a wide range of autoimmune disorders. ECP therapy also has an established safety profile. Real-world analyses demonstrate higher ECP continuation rates compared to other secondary systemic treatments [3], but its compliance with different types of intermittent fasting compared to *ad libitum* feeding behaviors has not been explored. This report aimed to describe the potential effects of Ramadan fasting on patients undergoing ECP.

## Case presentation

Patients undergoing ECP procedures were prospectively evaluated before and during Ramadan 1443 AH (2022 AD) at the Abu Dhabi Stem Cells Center (ADSCC), Abu Dhabi, United Arab Emirates. The following ECP outcomes were evaluated: treatment completion, adverse events reported, body mass index (BMI), and laboratory test results.

Laboratory testing included complete blood count (CBC), C-reactive protein (CRP), and systemic immune-inflammatory biomarkers (SIIBs): neutrophil-to-lymphocyte ratio (NLR), platelet-to-lymphocyte ratio (PLR), lymphocyte-to-monocyte ratio (LMR), and pan-immune-inflammation values (PIVs). PIVs were calculated as:

[Neutrophils (10^9^/L) × Platelets (10^9^/L) × Monocytes (10^9^/L)]/Lymphocytes (10^9^/L)

ECP procedures were performed using the Amicus^®^ Separator (Fresenius Kabi, Germany) and Therakos^®^ CellEx^®^ Photopheresis System (Mallinckrodt, Ireland) through central venous accesses.

The Common Terminology Criteria for Adverse Events (CTCAE v5.0) [4] were used to categorize peri-ECP complications. Pre-Ramadan parameters were considered to be the baseline (using the mean results of the previous four procedures).

Written informed consent was obtained from each participant, and the information was protected under the principles of confidentiality without revealing patients’ identities. Considering that the ECP procedures are part of standard clinical practices, data were reviewed and analyzed retrospectively, and no patients can be identified in this report; the Institutional Research Ethics Committee has confirmed the exemption from ethics review.

A total of three patients undergoing ECP at ADSCC were included in this report. All patients underwent treatment on an outpatient basis; one patient was treated using the Amicus^®^ Separator (Fresenius Kabi, Germany), and two patients were treated using the Therakos^®^ CellEx^®^ Photopheresis System (Therakos Inc., Mallinckrodt Pharmaceuticals, Ireland-United States). Central venous accesses were used for all patients, and ECP procedures were scheduled every two weeks before and during Ramadan. Patients’ demographics and outcomes are summarized in Tables 1-2, respectively.

**Table 1:** Demographics of patients undergoing ECP before and during Ramadan 1443 AH (2022 AD), Abu Dhabi (n=3) ^a^Fasting during the first ECP procedure during Ramadan ECP: extracorporeal photopheresis; cGvHD: chronic graft versus host disease

Patient	Gender	Age (years)	Diagnosis	Sessions before Ramadan	Sessions during Ramadan	Ramadan fasting	Ramadan treatment compliance	ECP platform
1	Female	36	cGvHD	31	1	No	No	Amicus^®^
2	Male	56	cGvHD	24	1	No	No	Therakos^®^ CellEx^®^
3	Male	41	pemphigus vulgaris	14	2	Yes ^a^	Yes	Therakos^®^ CellEx^®^

**Table 2:** Clinical and laboratory outcomes of patients undergoing ECP before and during Ramadan 1443 AH (2022 AD), Abu Dhabi (n=3) ^a^One-sample t-test *p-value statistically significant differences (p<0.05); **p-value statistically highly significant differences (p<0.01). Statistical analysis was performed using GraphPad^®^ Prism v8.4.3 (GraphPad^®^ Software Inc., USA) BMI: body mass index; CRP: C-reactive protein; Hg: hemoglobin, Hct: hematocrit; Plt: platelet count; Neu: neutrophils; Lym: lymphocytes; Mon: monocytes; NLR: neutrophil-to-lymphocyte ratio; PLR: platelet-to-lymphocyte ratio; LMR: lymphocyte-to-monocyte ratio; PIV: pan-immune-inflammation value

Parameter	Patient 1			Patient 2			Patient 3		
	Before	During	p^a^	Before	During	p^a^	Before	During	p^a^
BMI (kg/m^2^)	22.76	23.16	0.0030**	22.66	22.74	0.7108	18.61	18.62	0.5991
CRP (mg/L)	8.58	4.90	0.3001	34.08	37.70	0.7859	22.60	5.40	0.2674
Hg (g/dL)	11.85	11.90	0.8361	10.70	11.90	0.0580	10.93	10.90	0.5261
Hct (%)	34.95	37.00	0.1528	34.00	38.80	0.0209*	33.90	34.80	0.2750
Plt (10^9^/L)	267.75	268.00	0.9894	85.00	71.00	0.0963	247.75	375.00	0.0032**
Neu (10^9^/L)	6.17	7.22	0.2871	1.93	2.35	0.7548	4.07	1.38	0.1413
Lym (10^9^/L)	0.94	1.26	0.0684	0.30	0.25	0.3751	0.97	0.90	0.8231
Mon (10^9^/L)	0.57	0.40	0.0689	0.30	0.32	0.8225	0.51	0.34	0.2189
NLR	7.17	5.73	0.4685	8.21	9.40	0.4775	4.06	1.53	0.1022
PLR	300.22	212.70	0.1216	354.17	284.00	0.1313	266.74	416.67	0.0757
LMR	1.70	3.15	0.0167*	0.92	0.78	0.9085	2.01	2.65	0.0630
PIV	1147.65	614.27	0.1809	223.57	213.57	0.4274	531.56	195.50	0.1572

Patient 1 is a Muslim patient who was advised not to fast during Ramadan because she was classified as high-risk based on the BIMA recommendations. She required ECP to treat allogeneic stem cell transplantation complications (chronic GvHD). She did not fast during this month, and no new adverse events were reported. However, she could not comply with the treatment plan during Ramadan because she missed one session due to a mild COVID-19 infection, which did not require admission.

Patient 2 is a non-Muslim patient who was undergoing ECP to treat lung chronic GvHD, and no fasting was reported. Only one session was performed without incident or new adverse events, and his hematocrit significantly increased during the Ramadan procedure. His second procedure was deferred due to a grade 2 lung infection.

Patient 3 is a Muslim patient who was undergoing ECP to treat recalcitrant oral *pemphigus vulgaris*. Fasting was reported in his initial Ramadan procedure (day 3 of religious fasting), despite the advice to avoid fasting due to a low/moderate risk. During this session, the patient was fasting for more than eight hours, and we found a statistically significant increase in his platelet count (within a normal coagulation profile), grade 1 dehydration, and a vascular access complication (grade 2 blockage), which required treatment with tissue plasminogen activator, and the blockage was resolved. Changing the ECP mode from a double-needle to a single-needle modality was also required, increasing the procedure duration. The patient broke his fast for subsequent procedures, which were performed without incident.

## Discussion

Receiving the extra volume of intravenous solutions and medications during ECP does not invalidate the Ramadan fasting. The 10^th^ Conference of the Islamic Fiqh Council held in 1997 determined that injections do not affect the fast as long as they are administered for medical treatments and not for nutritional purposes [5,6]. However, an undetermined proportion of Muslim patients undergoing ECP may choose to fast.

The SIIB findings in our data correspond to the clinical effectiveness of ECP or the adverse events described for each patient. For instance, despite being tested negative for SARS-CoV-2 infection 24 hours before the ECP procedure, patient 1’s increased Ramadan LMR (p<0.05) was associated with an asymptomatic ongoing COVID-19 infection diagnosed three days after the procedure.

Our analyses showed that CRP levels decreased to normal values for patient 3 during Ramadan (no statistically significant difference), and it was higher than the result in the meta-analysis published by Allaf et al. (mean difference of −1.19 mg/L) [7]. A similar decrease in other SIIBs was observed in our non-fasting patients. We attributed it to the beneficial effects of ECP, which promote a tolerogenic dendritic cell phenotype, increase regulatory T cells, and diminish T cell activation [8,9].

In the same meta-analysis, the BMI of patients on intermittent fasting compared to *ad libitum* feeding (short term of ≤3 months) were compared, and there was a mean difference of −0.92 kg/m^2^ in favor of intermittent fasting, but it was not clinically significant [7]. This was not observed in our fasting patient, probably due to the short Ramadan fasting effects.

From our clinical experience, despite being well-nourished or hydrated, we recommend a comprehensive pre-Ramadan clinical assessment for all Muslim patients undergoing ECP, sharing current evidence on the effects of fasting and requesting advice on treatment plan changes by Muslim doctors. Alternatively, procedures scheduled during Ramadan can be rescheduled in the patient's best interest, if possible. These recommendations can improve their well-being and quality of life, resulting in better treatment compliance and clinical outcomes.

Our limitations are related to the small sample size and number of sessions during Ramadan. Additionally, decreasing overall SIIB tendencies are findings that were explained by the cumulative beneficial effects of previous ECP procedures, while the transient fasting effects are not sufficiently robust to explain the vascular access event in Patient 3. Other confounding effects in these three patients are related to the heterogeneity of the diagnoses, patient demographics, ECP platforms, and concomitant therapies.

## Conclusions

Although there are no age-, gender-, disease-specific, or other clinical or laboratory recommendations for accepting Ramadan fasting in ECP patients to date, we suggest avoiding fasting during photopheresis treatment (applicable to the intra- and inter-ECP procedures). This recommendation aligns with the fasting guidelines on the most common indications for diseases that require ECP treatment. Still, further studies with a larger sample size are needed to explain possible causality associations during the upcoming Ramadan months.
